# Editorial: Mineral nutrition and plant stress tolerance

**DOI:** 10.3389/fpls.2024.1461651

**Published:** 2024-07-25

**Authors:** Md Sazzad Hossain, Pedro Garcia Caparros, Karl Hermann Mühling

**Affiliations:** ^1^ Kiel University, Kiel, Germany; ^2^ Sylhet Agricultural University, Sylhet, Bangladesh; ^3^ University of Almeria, Almería, Spain

**Keywords:** plants, mineral elements, abiotic stress, biotic stress, plant physiology and biochemistry, nutrient deficiency and toxicity

The provision of so-called global food safety and security is threatened by global warming, climate change, and the increasing food demand for an ever-growing human population (Berkhout et al., 2019; Sun and Weaver, 2020; Dobermann et al., 2022). Improper plant nutritional management reduces crop production and quality becomes a vital global concern affecting billions of people worldwide (Kumssa et al., 2015; Hofmann et al., 2020; Dobermann et al., 2022). Any stress (biotic or abiotic) can disrupt plant metabolism and lead to reduced growth, fitness, and productivity (Hossain and Dietz, 2016; Ahmed et al., 2020). Understanding crop physiological and biochemical responses to adverse environmental conditions is critical (Bashir et al., 2021). Mineral nutrition is one of the most effective ways to reduce various stresses in crops to increase yield and quality. It plays a crucial role in the response of plants to both biotic and abiotic stresses (Marschner and Cakmak, 1989; Cakmak, 2005; Waraich et al., 2011; Marschner, 2012; Elmer and Datnoff, 2014; Cabot et al., 2019; Sarwar et al., 2019; Kumari et al., 2022). Interactions between mineral elements and biotic and abiotic stress responses are important for developing strategies to improve crop productivity and quality in stressed environments. Proper nutrient management can effectively mitigate the adverse effects of different stresses through diversified mechanisms (Shoukat et al., 2024a, b; Waraich et al., 2011; Mannan et al., 2022; Van Nguyen et al., 2022; Chowdhury et al., 2024).

Although considerable progress has been made in plant nutrition and stress tolerance many aspects of plant nutrition remain unknown. However, more extensive efforts are required to understand better the relationship between mineral elements and plant stress tolerance. Mechanisms underlying the role of mineral nutrition and its interactions with plants are proposed in this Research Topic, comprising diverse research articles, including two reviews and seven original research papers.

Nitrogen metabolism in crops is crucial for various physiological processes and plant growth, especially in staple crops like tea. In this review, Zhang et al. summarized the current information on the underlying mechanisms to identify key regulators in functional phenotypes and improve nitrogen use efficiency. The review highlighted the significance of ammonium as the primary nitrogen source. The biological and molecular mechanisms underlying the GS-GOGAT pathway, including nitrate reductase (NR), nitrite reductase (NiR), glutamine synthetase (GS), glutamate synthase (GOGAT), and glutamate dehydrogenase (GDH), were also explained in detail.

Phan et al. conducted a genome-wide association study to identify quantitative trait loci (QTLs) linked to nitrogen use efficiency (NUE) in rice under saline conditions. The research involved 2,391 rice accessions grown under two nitrogen conditions and two NaCl concentrations to assess dry weight. A total of 55 QTLs associated with the evaluated traits were identified, with 28 being novel discoveries. These findings offer valuable genetic resources for improving NUE in rice, particularly in saline environments.

Sugar and acid metabolism are pivotal in tomato development and fruit quality, necessitating further investigation into the underlying transcripts, particularly under high temperature and nitrogen fertilizer conditions. Zheng et al. reported that both conditions elevated the levels of soluble sugars and organic acids in young tomato fruits. Additionally, the study identified several genes involved in sucrose metabolism (CWINV2, HK2, SPS, PK) and sucrose transporters (SUT1, SUT4, SWEETs).

Chen et al. experimented to discern the main physiological and molecular mechanisms of *Acacia melanoxylon* stem in response to boron deficiency. Under boron-deficit conditions, stem growth was reduced with shortened internodes. Transcriptomic analysis revealed that genes linked to cell wall metabolism and structural components were downregulated. Furthermore, additional genes linked to hormone signaling showed significant alterations.

Moradi and Siosemardeh investigated the influence of seed priming and foliar application of various chemical fertilizers on rainfed wheat. Their study demonstrated that combining these application methods significantly enhanced the physiological and yield traits of the wheat. This information is crucial for growers seeking to improve plant growth and yield under drought-stressed conditions.

The review by Mukarram et al. focused on the interaction between silicon nanoparticles (SiNPs) and trace elements (TEs) toxicity. The authors emphasize exploring this interaction from an omics perspective, encompassing plant metabolomics, proteomics, and genomics. Furthermore, the review delves into the physiological and biochemical mechanisms underlying this interaction.

Pitann and Mühling examined the waterlogging resistance of oat at various developmental stages as an alternative for crop rotation in regions with temporary submergence. Their findings revealed that while late waterlogging negatively impacted the vegetative phase, it led to improved performance in the generative phase, resulting in increased grain yield. In contrast, early waterlogging severely affected oat performance during vegetative and generative phases.

Delgado et al. assessed the facilitation effects of *Gevuina avellana*, an aluminum hyperaccumulator, on the seedling growth and performance of *Vaccinium corymbosum*, a plant sensitive to aluminum intolerance and phosphorus deficiency, in soils supplemented with varying aluminum doses. The results indicated that co-cultivation with *G. avellana* ameliorated the growth conditions for *V. corymbosum*, highlighting the beneficial influence of *G. avellana*.

Luo et al. investigated the role of SPX-domain-containing proteins (SPXs) in phosphorus homeostasis in maize, with a particular emphasis on ZmSPX1. Their study demonstrated that overexpressed lines exhibited increased root sensitivity to both phosphorus deficiency and high-phosphorus conditions. These findings hold significant implications for enhancing phosphorus efficiency in maize breeding programs.

In conclusion, the articles included in this Research Topic contribute to our understanding of the efficacy of various nutrients in alleviating diverse stresses and plant nutrient relations, while illustrating the need for more such research. A better understanding of different nutrient elements could lead to more rational fertilizing practices, avoiding interactions that could contribute to the unbalanced mineral nutrition of plants for maximizing crop yield. This knowledge is also necessary to obtain more efficient genotypes in the acquisition of different nutrients.

## Author contributions

MH: Conceptualization, Writing – original draft, Writing – review & editing. PG: Writing – original draft, Writing – review & editing. KM: Supervision, Writing – original draft, Writing – review & editing.

## References

[B1] Ahmed M. Hasanuzzaman M. Raza M. A. Malik A. Ahmad S. (2020). Plant nutrients for crop growth, development and stress tolerance. Sustain. Agric. era Climate Change 1, 43–92. doi: 10.1007/978-3-030-45669-6_3

[B2] Bashir S. S. Hussain A. Hussain S. J. Wani O. A. Zahid Nabi S. Dar N. A.. (2021). Plant drought stress tolerance: Understanding its physiological, biochemical and molecular mechanisms. Biotech. Biotech. Equip. 35, 1912–1925. doi: 10.1080/13102818.2021.2020161

[B3] Berkhout E. D. Malan M. Kram T. (2019). Better soils for healthier lives? An econometric assessment of the link between soil nutrients and malnutrition in Sub-Saharan Africa. PLoS One 14, e0210642. doi: 10.1371/journal.pone.0210642 30653538 PMC6336299

[B4] Cabot C. Martos S. Llugany M. Gallego B. Tolrà R. Poschenrieder C. (2019). A role for zinc in plant defense against pathogens and herbivores. Front. Plant Sci. 10. doi: 10.3389/fpls.2019.01171 PMC6794951 31649687

[B5] Cakmak I. (2005). The role of potassium in alleviating detrimental effects of abiotic stresses in plants. J. Plant Nutr. Soil Sci. 168, 521–530. doi: 10.1002/jpln.200420485

[B6] Chowdhury M. S. N. Sani M. N. H. Siddique A. B. Hossain M. S. Yong J. W. H. (2024). Synergistic effects of biochar and potassium co-application on growth, physiological attributes, and antioxidant defense mechanisms of wheat under water deficit conditions. Plant Stress 12, 100452. doi: 10.1016/j.stress.2024.100452

[B7] Dobermann A. Bruulsema T. Cakmak I. Gerard B. Majumdar K. McLaughlin M.. (2022). Responsible plant nutrition: A new paradigm to support food system transformation. Glob Food Sec. 33, 100636. doi: 10.1016/j.gfs.2022.100636

[B8] Elmer W. H. Datnoff L. E. (2014). Mineral nutrition and suppression of plant disease. Encyclopedia of Agriculture and Food Systems. 4, 231–244. doi: 10.1016/B978-0-444-52512-3.00251-5

[B9] Hofmann T. Lowry G. V. Ghoshal S. Tufenkji N. Brambilla D. Dutcher J. R.. (2020). Technology readiness and overcoming barriers to sustainably implement nanotechnology-enabled plant agriculture. Nat. Food. 1, 416–425. doi: 10.1038/s43016-020-0110-1

[B10] Hossain M. S. Dietz K. J. (2016). Tuning of redox regulatory mechanisms, reactive oxygen species and redox homeostasis under salinity stress. Front. Plant Sci. 7. doi: 10.3389/fpls.2016.00548 PMC4861717 27242807

[B11] Kumari V. V. Banerjee P. Verma V. C. Sukumaran S. Chandran M. A. S. Gopinath K. A.. (2022). Plant nutrition: An effective way to alleviate abiotic stress in agricultural crops. Int. J. Mol. Sci. 23, 8519. doi: 10.3390/ijms23158519 35955651 PMC9368943

[B12] Kumssa D. B. Joy E. J. Ander E. L. Watts M. J. Young S. D. Walker S.. (2015). Dietary calcium and zinc deficiency risks are decreasing but remain prevalent. Sci. Rep. 5, 10974. doi: 10.1038/srep10974 26098577 PMC4476434

[B13] Mannan M. A. Tithi M. A. Islam M. R. Al Mamun M. A. Mia S. Rahman M. Z.. (2022). Soil and foliar applications of zinc sulfate and iron sulfate alleviate the destructive impacts of drought stress in wheat. Cer Res. Commun. 50, 1279–1289. doi: 10.1007/s42976-022-00262-5

[B14] Marschner P. (2012). Marschner’s Mineral Nutrition of Higher Plants. 3rd Edn (London: Academic Press).

[B15] Marschner H. Cakmak I. (1989). High light intensity enhances chlorosis and necrosis in leaves of zinc, potassium, and magnesium deficient bean (Phaseolus vulgaris) plants. J. Plant Physiol. 134, 308–315. doi: 10.1016/S0176-1617(89)80248-2

[B16] Sarwar M. Saleem M. F. Ullah N. Ali S. Rizwan M. Shahid M. R.. (2019). Role of mineral nutrition in alleviation of heat stress in cotton plants grown in glasshouse and field conditions. Sci. Rep. 9, 13022. doi: 10.1038/s41598-019-49404-6 31506449 PMC6737086

[B17] Shoukat A. Pitann B. Hossain M. S. Saqib Z. A. Nawaz A. Mühling K. H. (2024b). Zinc and silicon fertilizers in conventional and nano-forms: Mitigating salinity effects in maize (*Zea mays* L.). J. Plant Nutri Soil Sci. 1–12. doi: 10.1002/jpln.202300267

[B18] Shoukat A. Saqib Z. A. Akhtar J. Aslam Z. Pitann B. Hossain M. S.. (2024a). Zinc and silicon nano-fertilizers influence ionomic and metabolite profiles in maize to overcome salt stress. Plants 13, 1224. doi: 10.3390/plants13091224 38732438 PMC11085825

[B19] Sun H. Weaver C. M. (2020). Rise in potassium deficiency in the us population linked to agriculture practices and dietary potassium deficits. J. Agric. Food Chem. 68, 11121–11127. doi: 10.1021/acs.jafc.0c05139 32921052

[B20] Van Nguyen D. Nguyen H. M. Le N. T. Nguyen K. H. Nguyen H. T. Le H. M.. (2022). Copper nanoparticle application enhances plant growth and grain yield in maize under drought stress conditions. J. Plant Growth Regul. 41, 364–375. doi: 10.1007/s00344-021-10301-w

[B21] Waraich E. A. Ahmad R. Ashraf M. Y. (2011). Role of mineral nutrition in alleviation of drought stress in plants. Aust. J. Crop Sci. 5, 764–777. doi: 10.3316/informit.282340708899391

